# Salivary Oxytocin and Socio‐Emotional Functioning in Children and Adolescents With and Without Autism Spectrum Disorder

**DOI:** 10.1002/npr2.70138

**Published:** 2026-06-04

**Authors:** Minobu Ikehara, Rio Ishida, Kazuhiko Yamamuro, Natsuko Kashida, Tsutomu Takeda, Michihiro Toritsuka, Manabu Makinodan

**Affiliations:** ^1^ Department of Psychiatry Nara Medical University Nara Japan; ^2^ Department of Neuropsychiatry Kumamoto University Kumamoto Kumamoto Japan; ^3^ Center for Health Control Nara Medical University Nara Japan

**Keywords:** autism spectrum disorder, inflammatory cytokines, oxytocin, self‐esteem, social support

## Abstract

**Aim:**

Given that alterations in oxytocinergic and immune systems have been implicated in the social and emotional difficulties of autism spectrum disorder (ASD), yet the associations between these biological factors and internal psychological constructs remain unclear, we examined the relationship between salivary oxytocin (OT) levels, inflammatory markers (interleukin‐1*β* [IL‐1*β*], IL‐6, IL‐8), self‐esteem, and perceived social support in children and adolescents with and without ASD.

**Methods:**

26 children and adolescents with ASD and 23 typically developing (TD) controls were included. Salivary OT and inflammatory cytokine levels were measured using enzyme‐linked immunosorbent and multiplex immunoassays.

**Results:**

Salivary oxytocin levels were significantly lower in the ASD group than in the TD group after adjusting for age. In contrast, no significant group differences were observed in inflammatory markers (IL‐1*β*, IL‐6, or IL‐8). However, across participants, including those with ASD, higher OT levels were associated with greater self‐esteem and perceived social support. These findings suggest that oxytocin‐related socio‐emotional processes may be associated with socio‐emotional functioning across children and adolescents, including those with ASD. Participants with higher OT levels were associated with greater self‐esteem and perceived social support. Among the inflammatory markers, none of the cytokines (IL‐1*β*, IL‐6, or IL‐8) showed significant associations with the psychological measures, including AQ‐J total scores.

**Conclusion:**

OT levels may be associated with socio‐emotional functioning and psychological well‐being across children and adolescents with and without ASD, regardless of diagnostic status. In contrast, no significant associations were observed between inflammatory markers and psychological measures in the present sample.

## Abstract 2

Salivary oxytocin levels were associated with self‐esteem and perceived social support across children and adolescents with and without ASD, whereas inflammatory markers showed no significant associations. These findings suggest that oxytocin‐related processes may contribute to socio‐emotional functioning regardless of diagnostic status.

## Graphical abstract

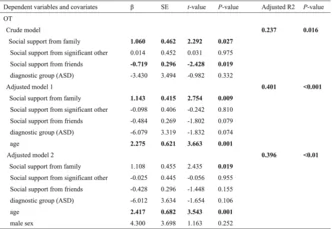

## 1 Introduction

Autism spectrum disorder (ASD) is a complex neurodevelopmental condition that affects 1 in 54 children worldwide. It is mainly characterized by persistent deficits in social communication, restricted interests, and repetitive behaviors (*Diagnostic and Statistical Manual of Mental Disorders: DSM‐5‐TR*). Individuals with ASD frequently experience comorbid conditions, such as anxiety, depression, and difficulties with emotional regulation, which significantly impact their daily functioning and quality of life [1, 2].

The potential role of oxytocin (OT), a neuropeptide involved in social bonding, emotional regulation, and affiliative behavior, in social functioning and socio‐emotional processes has been highlighted [3, 4]. Lower peripheral OT levels have been reported in individuals with ASD in several studies, suggesting that dysregulation of the oxytocinergic system may contribute to the social impairments observed in this population [5, 6, 7]. However, findings remain inconsistent, as some studies have shown that OT levels do not significantly differ between individuals with ASD and typically developing (TD) controls [6, 8]. Given the role of OT in modulating social behaviors [9, 10, 11, 12, 13, 14], further research is necessary to clarify its effects on psychological factors, such as self‐esteem and perceived social support, in individuals with ASD.

The role of immune dysfunction and chronic inflammation in ASD has been increasingly investigated in recent years. Studies have reported altered levels of interleukin‐6 (IL‐6), IL‐8, and IL‐1*β* in individuals with ASD [15, 16, 17, 18, 19]. Neuroinflammation reportedly influences synaptic function, neural plasticity, and behavior, all of which are important in ASD symptomatology [20, 21, 22]. Most previous studies investigating immune alterations in ASD have measured inflammatory cytokines in peripheral blood. However, saliva sampling offers a non‐invasive and stress‐free method that is particularly suitable for studies involving children and adolescents. Salivary cytokines have been increasingly used in psychoneuroimmunology research and may reflect peripheral inflammatory activity to some extent. Therefore, assessing inflammatory markers in saliva may provide a practical approach to examining immune‐related processes in children and adolescents [15, 23].

The roles of OT and inflammatory markers in ASD have been investigated in previous studies; however, several critical gaps remain. OT influences social cognition and emotional regulation; however, its relationship with self‐esteem in ASD populations has largely been overlooked. The direct impact of OT on social behaviors has been the focus of most research [4, 6], neglecting its potential role in shaping internal psychological constructs such as self‐worth and perceived social support. Given that individuals with ASD often report low self‐esteem [24], understanding the link between OT and self‐esteem could offer valuable insights into the mechanisms underlying socio‐emotional difficulties. Additionally, although immune dysregulation is commonly observed in ASD [17, 25, 26, 27], the functional consequences of elevated inflammatory markers on social behavior and psychological well‐being are not fully understood. Altered levels of inflammatory cytokines, including IL‐6, IL‐8, and IL‐1*β*, have been reported in individuals with ASD [15, 16, 17, 18, 19]; meanwhile, the functional consequences of these immune alterations remain unclear. Chronic inflammation has been previously demonstrated in ASD [26, 28, 29, 30]; however, its impact on self‐esteem, social support, and emotional resilience remains underexamined. Clarifying these associations could help identify the biological pathways that influence social and emotional functioning associated with ASD.

In the present study, socio‐emotional functioning is operationally defined as a multidimensional construct encompassing self‐esteem and perceived social support, reflecting individuals' subjective evaluation of themselves and their social relationships. By integrating biological and psychological measures, we aimed to provide a more comprehensive understanding of how neuroendocrine and immune factors relate to self‐perception and social experiences in children and adolescents. In this context, socio‐emotional functioning refers to internal psychological constructs, such as self‐esteem and perceived social support, which are important for emotional well‐being and adaptive social engagement in both individuals with ASD and typically developing populations.

## 2 Methods

### 2.1 Participants

This study included 26 children and adolescents with ASD and 23 TD controls aged 8–17 years, who were born and raised in Japan. Individuals in the ASD group were recruited from the outpatient services of the Department of Psychiatry at Nara Medical University Hospital and its affiliated hospitals. ASD diagnoses were established by two trained psychiatrists based on the DSM‐5‐TR criteria (*Diagnostic and Statistical Manual of Mental Disorders: DSM‐5‐TR*) and were further confirmed using the Autism Diagnostic Observation Schedule‐2 [31].

Cognitive functioning was assessed using the Das–Naglieri Cognitive Assessment System (DN‐CAS), a theory‐driven instrument used to evaluate four cognitive processing domains: planning, attention, simultaneous processing, and successive processing [32]. Participants with a total DN‐CAS score < 70 were excluded to minimize the influence of intellectual disability, following previous reports that a score < 70 indicates significant cognitive impairment [32]. Individuals with major neurological disorders were excluded. Participants with ASD were not excluded on the basis of psychiatric comorbidities or psychotropic medication use. Detailed information on psychiatric comorbidities and psychotropic medication use is provided in Tables S1 and S2.

This study was approved by the Ethics Committee of Nara Medical University (Approval Nos. 1319, 2535). Because all participants were minors (aged 8–17 years), written informed consent was obtained from their parents or legal guardians, and assent was obtained from the participants whenever possible, in accordance with the Declaration of Helsinki.

### 2.2 Saliva Collection and Biomarker Analysis

Saliva samples were collected using the passive drool method following the standardized protocols established by The Vanderbilt Hormone Assay & Analytical Services Core and Salimetrics Saliva Lab (Carlsbad, CA, USA). Participants were instructed to avoid brushing their teeth for at least 45 min before sample collection and to refrain from undergoing any dental procedures within 24 h. The samples were stored at −80°C and later shipped on dry ice to the Salimetrics laboratory for analysis.

Salivary OT concentrations were measured using enzyme‐linked immunosorbent assays validated for human saliva. Samples were assayed for the Salimetrics Cytokine Panel (IL‐1*β*, IL‐6, TNF‐α, and IL‐8) in duplicate at the Salimetrics SalivaLab (Carlsbad, CA) using a proprietary electrochemiluminescence method developed and validated for saliva by Salimetrics. The average coefficient of variation for all samples tested was < 15%, which meets the SalivaLab's criteria for accuracy and repeatability in Salivary Bioscience, and exceeds the applicable NIH guidelines for Enhancing Reproducibility through Rigor and Transparency. Sample test volume was 25 μL of saliva per determination. The IL‐1*β* assay has a lower limit of sensitivity of 0.05 pg/mL, with a dynamic range from 0.05–2256 pg/mL. Before testing, samples were stored at −80°C before being shipped on dry ice to the Salimetrics SalivaLab.

### 2.3 Psychological Assessments

Three standardized instruments were used to assess psychological characteristics: the Rosenberg Self‐Esteem Scale (RSES), Multidimensional Scale of Perceived Social Support (MSPSS), and Japanese version of the Autism‐Spectrum Quotient (AQ‐J). The AQ‐J was originally developed for adults; nonetheless, we adopted an age‐appropriate administration method: participants aged ≥ 16 years completed the AQ‐J by self‐report, while parents or caregivers completed it on behalf of children aged < 16 years, following similar modifications in previous studies [33]. Moreover, although the RSES and MSPSS were designed for adolescents and adults, they have been successfully used in younger populations in previous studies, provided that sufficient explanation and adult assistance were given during administration [34, 35]. For participants who were < 12 years, caregivers supported questionnaire comprehension as needed, ensuring appropriate understanding and response validity.

### 2.4 RSES

The RSES is a widely used 10‐item scale assessing global self‐esteem. Items are rated on a 4‐point Likert scale from “strongly disagree” to “strongly agree,” with higher scores indicating greater self‐esteem. The Japanese version, validated by Yamamoto et al., demonstrates good internal consistency (Cronbach's α = 0.81) and construct validity [34, 36]. This measure was selected for its reliability and sensitivity in assessing self‐esteem in individuals with ASD.

### 2.5 MSPSS

The MSPSS is a 12‐item scale assessing perceived social support from family, friends, and significant others [35, 37]. Items are rated on a 7‐point Likert scale, with higher scores indicating greater perceived social support. The Japanese version has demonstrated high reliability (Cronbach's α = 0.85–0.91) and was used to assess perceived social support in children and adolescents with ASD.

### 2.6 AQ‐J

The AQ‐J is a 50‐item questionnaire assessing autistic traits across five domains: social skills, attention switching, attention to detail, communication, and imagination. Items are rated on a 4‐point Likert scale, with higher scores indicating greater autistic traits. The Japanese version shows good internal consistency (Cronbach's α = 0.77–0.86) [33]. Participants aged ≥ 16 years completed the self‐report version, whereas parents or caregivers completed the assessment for those < 16 years.

### 2.7 Statistical Analysis

Statistical analyses were conducted using IBM SPSS Statistics (version 28; IBM Corp., Armonk, NY, USA) and R software (version 4.5.1; R Foundation for Statistical Computing, Vienna, Austria) for rank‐based robust regression analyses. Continuous variables are presented as mean, median, and interquartile range (IQR), whereas categorical variables are presented as counts and percentages. Group differences in demographic variables were examined using the two‐sided Mann–Whitney U test for continuous variables and Pearson's chi‐square test for categorical variables. Effect sizes for Mann–Whitney U tests were calculated as r = |Z|/√N. To examine group differences in biological and psychological measures while accounting for age differences between groups, rank‐based robust regression analyses (nonparametric regression) were performed with diagnostic group (ASD vs. TD) as the predictor and age included as a covariate. Associations between salivary biomarkers (oxytocin [OT], interleukin‐1*β* [IL‐1*β*], IL‐6, and IL‐8) and psychological measures (Rosenberg Self‐Esteem Scale [RSES], Multidimensional Scale of Perceived Social Support [MSPSS], and Autism‐Spectrum Quotient–Japanese version [AQ‐J]) were examined using nonparametric partial correlations (Spearman's partial correlation) while controlling for age. Analyses were conducted across the combined sample of ASD and TD participants. To account for multiple testing, *P* were adjusted using the Benjamini–Hochberg false discovery rate (FDR) procedure. For MSPSS subscales, FDR correction was applied across 16 tests (four biomarkers × four social support measures), whereas for other psychological measures, correction was applied across four tests (four biomarkers per measure). Associations with FDR‐adjusted *p* < 0.05 were considered statistically significant.

To further examine the relationship between perceived social support and salivary OT concentrations, rank‐based robust regression analyses were performed with OT as the dependent variable and diagnostic group (ASD vs. TD) together with the three MSPSS subscales (family, significant other, and friends) as independent variables. Additional models were adjusted for age (Model 1) and for both age and sex (Model 2). Logistic regression analyses were conducted to evaluate predictors of ASD diagnosis. Four models were constructed: a crude model without covariates; Adjusted Model 1, controlling for age and sex; Adjusted Model 2, additionally including salivary OT levels; and Adjusted Model 3, including AQ‐J total scores alongside age and sex. Odds ratios (ORs) with 95% confidence intervals (CIs) were calculated to estimate the strength of associations. All *P* were two‐sided, and statistical significance was set at *p* < 0.05 unless otherwise specified. To clarify the analytical framework, statistical analyses were conducted in a hierarchical manner. First, age‐adjusted group comparisons were performed to examine differences between ASD and TD groups in biological and psychological variables (Table 2). Second, age‐adjusted partial correlations were conducted across the combined sample to explore associations between salivary biomarkers and psychological measures (Table 3). Third, multivariable robust regression analyses were performed to identify independent predictors of salivary oxytocin levels, including diagnostic group and perceived social support (Table 4), and these models were considered the primary analyses. Finally, logistic regression analyses were conducted to examine predictors of ASD diagnosis. Group comparisons and partial correlations were treated as descriptive or exploratory analyses.

## 3 Results

### 3.1 Demographic and Biomarker Differences

Table 1 presents the demographic characteristics of the TD and ASD groups. The ASD group was significantly older than the TD group (*p* = 0.009). The sex distribution did not significantly differ between the groups (*p* = 0.807). The comparisons of biological and psychological measures between the groups are summarized in Table 2. Robust regression analyses adjusting for age showed that salivary OT levels were significantly lower in the ASD group than in the TD group (*p* = 0.005). In contrast, no significant group differences were observed for the inflammatory markers IL‐1*β* (*p* = 0.846), IL‐6 (*p* = 0.940), or IL‐8 (*p* = 0.634). Regarding psychological measures, the ASD group showed significantly lower scores on the Rosenberg Self‐Esteem Scale (*p* = 0.023) and the Multidimensional Scale of Perceived Social Support total score (*p* = 0.035). Among the MSPSS subscales, perceived family support was significantly lower in the ASD group (*p* = 0.006), whereas support from significant others (*p* = 0.154) and friends (*p* = 0.125) did not significantly differ between the groups. As expected, AQ‐J scores were significantly higher in the ASD group than in the TD group (*p* < 0.001). No significant group difference was observed in DN‐CAS full‐scale scores (*p* = 0.871).

**TABLE 1:** Demographic characteristics of children and adolescents in TD and ASD groups.

	TD			ASD					
	*N* = 23			*N* = 26					
	average	median	IQR	average	median	IQR	U	*p*	r
Age (year)	11.96	12.00	10.00–14.00	13.88	15.00	12.00–15.00	**446.500**	**0.009**	**0.371**
Sex (male/female)	17/6			20/6				0.807	

*Note:* Data are presented as median and interquartile range (IQR) unless otherwise indicated. Group differences between the autism spectrum disorder (ASD) and typically developing (TD) groups were examined using the two‐sided Mann–Whitney U test. Sex distribution was compared using Pearson's chi‐square test (χ^2^ = 0.060, *p* = 0.807). Effect size (r) was calculated as |Z|/√N. Bold values indicate statistical significance at *p* < 0.05.

Abbreviations: ASD, autism spectrum disorder; IQR, interquartile range; r, effect size; TD, typically developing; U, Mann–Whitney U statistic.

**TABLE 2:** Comparison of biological and psychological measures between TD and ASD groups.

	TD			ASD				
	*N* = 23			*N* = 26				
	average	median	IQR	average	median	IQR	Estimate (*β*)	*p*
OT	17.92	16.48	10.58–20.64	15.70	7.32	3.94–26.67	**−8.793**	**0.005**
IL‐I*β*	94.55	83.12	63.23–105.40	133.00	82.68	62.72–177.55	3.322	0.846
IL‐6	5.15	4.82	3.06–6.49	9.65	3.69	2.78–11.44	0.070	0.940
IL‐8	737.57	750.34	622.80–859.78	1301.29	662.64	443.85–1156.46	−55.258	0.634
RSES	27.78	28.00	25.00–32.00	23.58	21.50	19.00–29.00	**−5.00**	**0.023**
MSPSS								
Social support total	67.61	69.00	55.50–80.00	54.88	60.00	45.00–68.00	**−10.57**	**0.035**
Social support from family	23.61	25.00	20.50–27.00	19.27	21.00	16.00–23.00	**−4.00**	**0.006**
Social support from significant other	22.35	22.00	18.00–27.50	18.77	20.50	13.00–24.00	−3.00	0.154
Social support from friends	21.65	23.00	17.50–26.00	16.85	19.00	11.00–23.00	−3.40	0.125
AQ‐J	14.87	17.00	10.00–20.00	28.15	28.00	22.00–34.00	**12.67**	**< 0.001**
DN‐CAS	96.87	101.00	89.00–104.50	98.46	97.50	86.00–108.00	0.78	0.871

*Note:* Values are presented as mean, median, and interquartile range (IQR). Group differences were examined using robust regression analyses (rank‐based regression) with diagnostic group as the predictor and age included as a covariate. *β* represents the regression coefficient for the ASD group relative to the TD group. Bold values indicate statistical significance at *p* < 0.05.

Abbreviations: AQ‐J, Autism Spectrum Quotient–Japanese version; DN‐CAS, Das–Naglieri Cognitive Assessment System; IL‐1*β*, interleukin‐1 beta; IL‐6, interleukin‐6; IL‐8, interleukin‐8; IQR, interquartile range; MSPSS, Multidimensional Scale of Perceived Social Support; OT, oxytocin; RSES, Rosenberg Self‐Esteem Scale.

### 3.2 Associations Between Oxytocin, Self‐Esteem, and Social Support

As shown in Table 3, age‐adjusted nonparametric partial correlation analyses revealed that salivary OT levels were positively associated with perceived family support (*p* < 0.001). OT levels were also positively associated with self‐esteem (*p* < 0.05). In contrast, the associations between OT and total social support, support from significant others, support from friends, and AQ‐J scores were not statistically significant. Regarding inflammatory markers, none of the cytokines (IL‐1*β*, IL‐6, or IL‐8) showed significant correlations with psychological measures after FDR correction.

**TABLE 3:** Age‐adjusted partial correlations between salivary biomarkers and psychological test scores.

	OT	IL‐1*β*	IL‐6	IL‐8
MSPSS				
Social support total	0.338	0.030	−0.033	0.075
Social support from family	0.535***	0.065	0.069	0.091
Social support from significant other	0.312	−0.021	−0.074	0.102
Social support from friends	0.100	0.025	−0.087	−0.100
Rosenberg self‐esteem scale total	0.358*	0.047	0.010	0.134
AQ‐J total	−0.274	0.300	0.180	0.148

*Note:* Partial correlation coefficients between salivary biomarkers and psychological test scores are shown. Nonparametric partial correlations (Spearman's partial correlation) were calculated after controlling for age. *p*‐values were adjusted for multiple comparisons using the Benjamini–Hochberg false discovery rate (FDR) procedure. For the Multidimensional Scale of Perceived Social Support (MSPSS), FDR correction was applied across 16 tests (4 salivary biomarkers × 4 social support measures). For the other psychological measures, FDR correction was applied across four tests (4 biomarkers per measure). Bold values indicate statistical significance at *p* < 0.05. Only associations with FDR‐adjusted *p* < 0.05 were considered statistically significant. *p < 0.05, ***p < 0.001.

Abbreviations: AQ‐J, Autism‐Spectrum Quotient–Japanese version; FDR, false discovery rate; IL‐1*β*, interleukin‐1 beta; IL‐6, interleukin‐6; IL‐8, interleukin‐8; MSPSS, Multidimensional Scale of Perceived Social Support; OT, oxytocin.

### 3.3 Associations between Oxytocin and Perceived Social Support

To further examine the relationship between salivary OT levels and perceived social support, we conducted robust regression analyses with salivary OT concentration as the dependent variable and diagnostic group (ASD vs. TD) together with the three MSPSS subscales as independent variables (Table 4). In the crude model, perceived family support was positively associated with OT levels (*p* = 0.027), whereas support from friends showed a negative association with OT levels (*p* = 0.019). Support from significant others was not significantly associated with OT levels (*p* = 0.975). Diagnostic group (ASD vs. TD) was not significantly associated with OT levels in the crude model (*p* = 0.332). After adjusting for age (adjusted model 1), family support remained significantly associated with OT levels (*p* = 0.009), whereas support from significant others and friends were not significant predictors. Age showed a significant positive association with OT levels (*p* = 0.001), whereas diagnostic group remained non‐significant (*p* = 0.074). Similar results were observed after further adjustment for sex (adjusted model 2), where family support remained significantly associated with OT levels (*p* = 0.019). Age remained a significant predictor (*p* = 0.001), whereas sex (*p* = 0.252) and diagnostic group (*p* = 0.106) were not significantly associated with OT levels. Overall, these findings indicate that perceived family support was consistently associated with salivary OT levels across the regression models. An additional regression analysis including the interaction term between diagnostic group and family support did not reveal a significant interaction (*p* = 0.18), indicating that the association between family support and salivary oxytocin did not significantly differ between ASD and TD participants. Importantly, no significant interaction effects were observed, suggesting that the association between oxytocin and socio‐emotional measures was not specific to ASD diagnosis.

**TABLE 4:** Robust regression analyses of the association between perceived social support and salivary oxytocin concentration.

Dependent variables and covariates	*β*	SE	*t*	*p*	Adjusted *R*2	*p*
OT						
Crude model					**0.237**	**0.016**
Social support from family	**1.060**	**0.462**	**2.292**	**0.027**		
Social support from significant other	0.014	0.452	0.031	0.975		
Social support from friends	**−0.719**	**0.296**	**−2.428**	**0.019**		
diagnostic group (ASD)	−3.430	3.494	−0.982	0.332		
Adjusted model 1					**0.401**	**< 0.001**
Social support from family	**1.143**	**0.415**	**2.754**	**0.009**		
Social support from significant other	−0.098	0.406	−0.242	0.810		
Social support from friends	−0.484	0.269	−1.802	0.079		
diagnostic group (ASD)	−6.079	3.319	−1.832	0.074		
age	**2.275**	**0.621**	**3.663**	**0.001**		
Adjusted model 2					**0.396**	**< 0.01**
Social support from family	1.108	0.455	2.435	0.019		
Social support from significant other	−0.025	0.445	−0.056	0.955		
Social support from friends	−0.428	0.296	−1.448	0.155		
diagnostic group (ASD)	−6.012	3.634	−1.654	0.106		
age	**2.417**	**0.682**	**3.543**	**0.001**		
male sex	4.300	3.698	1.163	0.252		

*Note:* The dependent variable was salivary oxytocin concentration (pg/mL). Independent variables were perceived diagnostic group (ASD vs. TD) and social support subscales derived from the Multidimensional Scale of Perceived Social Support. Associations were examined using rank‐based robust regression (nonparametric regression). The crude model included social support variables only. Adjusted model 1 was additionally adjusted for age, and adjusted model 2 was further adjusted for age and male sex as covariates. Variance inflation factor (VIF) values were < 3.2, indicating no serious multicollinearity. Multiple regression analyses were conducted to examine the associations between salivary oxytocin levels and social support, diagnostic group, age, and sex. Family support was consistently positively associated with oxytocin levels across the models, while support from friends showed a negative association in the crude model. Age was also positively associated with oxytocin levels after adjustment. In contrast, diagnostic group and sex were not significantly associated with oxytocin levels in the adjusted models. Bold values indicate statistical significance at *p* < 0.05.

Abbreviations: OT, oxytocin; *R* ^2^, coefficient of determination; SE, standard error; VIF, variance inflation factor.

### 3.4 Predictors of ASD Diagnosis

As shown in Table 5, the AQ‐J total score consistently predicted ASD diagnosis in all models. In the crude model, higher AQ‐J scores were associated with greater odds of ASD (OR = 1.581, *p* = 0.003), whereas OT was not significant (OR = 0.989, *p* = 0.588). In Adjusted Model 1, which controlled for age and sex, the AQ‐J remained a significant predictor (OR = 1.584, *p* = 0.003). In this model, OT showed a trend toward significance (OR = 0.953, *p* = 0.061), but did not reach the conventional threshold. In Adjusted Model 2, which included the AQ‐J total score while adjusting for age, sex, and salivary OT, the AQ‐J remained significant (OR = 1.568, *p* = 0.004). Finally, in Adjusted Model 3, in which OT was examined while controlling for age, sex, and AQ‐J, OT was not a significant predictor (OR = 0.985, *p* = 0.688).

**TABLE 5:** Logistic regression models predicting ASD diagnosis.

AQ‐J	Odds ratio (95% CI)	*p*
Crude model	1.581 (1.164–2.148)	**0.003**
Adjusted model 1^a^	1.584 (1.168–2.148)	**0.003**
Adjusted model 2^b^	1.568 (1.151–2.137)	**0.004**
Oxytocin		
Crude model	0.989 (0.951–1.029)	0.588
Adjusted model 1^a^	0.953 (0.906–1.002)	0.061
Adjusted model 3^c^	0.985 (0.918–1.058)	0.688

*Note:* Bold values indicate statistical significance at *p* < 0.05.

Abbreviations: AQ‐J, Japanese version of the Autism‐Spectrum Quotient; CI, confidence interval.

^a^ Model a was adjusted for age and sex.

^b^ Model b was adjusted for age, sex, and salivary oxytocin.

^c^ Model c was adjusted for age, sex, and AQ‐J total score *p*‐values represent tests of the regression coefficients for each predictor, indicating whether the odds ratio differs from 1.

## 4 Discussion

We examined the relationships between salivary OT levels, inflammatory markers, self‐esteem, and social support in children and adolescents with and without ASD. These psychological constructs are considered core components of socio‐emotional functioning, which encompasses emotional resilience and the ability to form and perceive supportive social relationships. Regarding oxytocin, the findings revealed that higher OT levels were significantly associated with greater perceived social support. Higher OT levels were also associated with greater self‐esteem and perceived social support, including family support. Therefore, OT levels may be associated with socio‐emotional well‐being regardless of diagnostic status. In age‐adjusted group comparisons, salivary OT levels were significantly lower in the ASD group than in the TD group. However, in multivariable regression analyses including perceived social support and covariates, the diagnostic group was not an independent predictor of OT levels. These findings suggest that although group differences were observed descriptively, OT levels may be more strongly associated with socio‐emotional factors than with diagnostic status itself. Similarly, levels of IL‐1*β*, IL‐6, and IL‐8 did not significantly differ between the ASD and TD groups, and none of these inflammatory markers showed significant associations with psychological measures.

OT levels were significantly correlated with internal psychological variables such as perceived social support, suggesting that the oxytocinergic system may be associated with social behavior and certain aspects of psychological well‐being [38, 39, 40, 41]. Previous studies have yielded inconsistent results; nonetheless, some reports have shown that OT levels do not significantly differ between individuals with ASD and TDs [8]. These findings contribute to this debate by revealing that, although OT levels did not significantly differ between the groups, they were significantly associated with the quality of supportive interpersonal relationships across participants. Collectively, the current results underscore the relevance of OT levels to socio‐emotional functioning, particularly in relation to perceived support from family members. This perspective aligns with the existing literature, which highlights the role of OT in social bonding and emotional regulation. Reduced peripheral OT levels have been reported in ASD [5, 6, 7]; however, findings across studies remain inconsistent. The present results suggest that the functional role of OT may be more important than its peripheral concentration in understanding its relationship with psychological well‐being. In contrast, OT levels were not significantly associated with perceived support from friends or autistic traits, as measured using the AQ‐J, after correction for multiple comparisons. Although nominal associations were observed before correction, the lack of statistical significance indicates that OT may be more closely related to specific interpersonal contexts than to broader psychological characteristics. These findings suggest that oxytocin‐related socio‐emotional functioning may be differentially associated with close interpersonal contexts, particularly family relationships, rather than broader social domains.

Additional regression analyses further indicated that family support was the only subscale of the MSPSS that showed a significant association with salivary OT, whereas support from friends and significant others did not. This pattern may reflect that OT‐related socio‐emotional processes in children and adolescents are more closely linked to the family environment than to other support sources. Because the analyses included participants with ASD and TDs, this association should be interpreted as a general pattern across participants rather than as a diagnosis‐specific effect. Although the associations were observed across the combined sample, the findings may provide insight into socio‐emotional functioning in both ASD and typically developing populations. In the present study, the associations between oxytocin levels and socio‐emotional measures were observed across the combined sample, suggesting that oxytocin‐related processes may be associated with socio‐emotional functioning across participants, including individuals with ASD. Thus, even though the associations were not diagnosis‐specific, the findings may reflect dimensional associations between oxytocin and socio‐emotional functioning across participants. Collectively, the findings reveal that family context may play an important role in understanding how OT relates to socio‐emotional functioning. A novel finding of this study was the significant association between OT levels and self‐esteem. Low self‐esteem is frequently reported in individuals with ASD [24]; however, few studies have explored its neurobiological basis. Given the involvement of OT in emotional regulation, stress buffering, and social reward processing, higher OT levels may be related to a more positive self‐concept and greater perceived support. These findings suggest that OT may be relevant to psychological resilience and socio‐emotional functioning. However, further studies are required to determine whether OT could serve as a biomarker or therapeutic target. In addition to oxytocin, we also examined inflammatory cytokines. Previous studies have reported altered cytokine levels among individuals with ASD, suggesting that chronic neuroinflammation may contribute to ASD symptomatology [15, 16, 17, 18, 19]. However, in the present study, none of the inflammatory markers (IL‐1*β*, IL‐6, or IL‐8) showed significant associations with psychological measures. These findings suggest that the relationship between inflammatory markers and socio‐emotional or behavioral characteristics may be complex and requires further investigation. In contrast, OT levels were associated with internal psychological dimensions, including self‐esteem and perceived social support.

Regarding inflammatory markers, no significant associations were observed between IL‐1*β*, IL‐6, or IL‐8 and psychological measures in the present study. Previous studies have reported altered cytokine levels in individuals with ASD, suggesting that immune dysregulation and neuroinflammation may be involved in ASD‐related processes [15, 16, 17, 18, 19]. However, the current findings did not detect significant relationships between these inflammatory markers and autistic traits or socio‐emotional measures, indicating that the influence of inflammatory cytokines on psychological characteristics may be complex and require further investigation. Previous studies have primarily measured inflammatory cytokines in peripheral blood. Although the present study did not identify significant cytokine‐related associations, saliva sampling remains a non‐invasive approach that may be useful for investigating immune‐related processes in pediatric populations.

The findings also have implications for future OT‐based interventions targeting socio‐emotional functioning. Several clinical trials have explored the effects of intranasal OT on social cognition and behavior [41, 42, 43, 44, 45]; however, the results are inconsistent, with some studies showing improvements and others reporting no significant effects [9, 11, 46]. One possible explanation for these inconsistencies is the narrow focus on observable social behaviors, often neglecting internal states such as self‐esteem and emotional resilience. Our results show that OT may be related to broader aspects of psychological well‐being that may not be fully captured by conventional outcome measures. Comprehensive evaluation of oxytocin‐based interventions would benefit from incorporating assessments of both external social behaviors and internal psychological functioning. Additionally, combining OT administration with psychosocial interventions—such as social skills training, caregiver involvement, and cognitive‐behavioral therapy—may enhance treatment outcomes. Given the significant association observed between OT levels and perceived family support, family‐based therapeutic approaches may be especially effective in leveraging the potential benefits of OT. OT levels were not significant predictors of ASD diagnosis in the logistic regression models; nonetheless, the findings suggest that OT may still contribute as a complementary factor when combined with psychological measures. Hence, further research is needed to determine whether salivary OT could serve as an adjunctive indicator, particularly in the context of multidimensional assessments of ASD. Although the associations between oxytocin levels and perceived family support were observed across the combined sample, these findings may provide insight into socio‐emotional functioning in both ASD and typically developing populations. Individuals with ASD often experience reduced perceived social support and difficulties in family and social relationships. The present findings suggest that oxytocin‐related socio‐emotional processes may be associated with socio‐emotional functioning across participants, including individuals with ASD. Because the observed associations were identified across the combined sample and interaction effects were not significant, the present findings should be interpreted as dimensional rather than ASD‐specific associations.

Despite its contributions, this study has some limitations. First, the sample size was relatively small, potentially limiting the statistical power to detect group differences and interaction effects. In addition, because the associations were identified across the combined sample, the findings should not be interpreted as evidence of an ASD‐specific mechanism. Second, the cross‐sectional design precluded causal inferences regarding the directionality of the observed associations. Longitudinal studies are needed to determine whether changes in OT levels precede or result from improvements in psychological functioning. Third, although salivary OT is a non‐invasive proxy for peripheral OT, its relationship with central OT activity remains debated [44]. Future research should incorporate neuroimaging, cerebrospinal fluid analysis, and genetic approaches to comprehensively understand the role of OT in ASD. Fourth, no significant associations were observed between inflammatory markers and psychological measures in the present study, and the functional significance of these cytokines remains unclear. The relevance of inflammatory markers to psychological interventions remains to be established. Finally, although the MSPSS and RSES were originally developed for adolescents and adults, they were administered to younger children with caregiver assistance to ensure comprehension. Caregiver involvement, particularly in younger participants, may have influenced responses to the MSPSS family subscale and potentially led to an overestimation of perceived family support. This potential response bias should be considered when interpreting the findings.

In conclusion, the findings of this study highlight the potential relevance of OT to socio‐emotional functioning in children and adolescents. Salivary OT levels were significantly associated with self‐esteem and perceived social support across participants, suggesting that OT may be associated with socio‐emotional functioning and psychological well‐being regardless of diagnostic status. Thus, salivary oxytocin may serve as a biologically relevant indicator associated with socio‐emotional functioning, including self‐esteem and perceived social support, across children and adolescents with and without ASD, although the associations were not diagnosis‐specific. Future research should further investigate the role of oxytocinergic processes and consider integrative approaches that combine biological and psychosocial strategies to better understand socio‐emotional functioning.

## Author Contributions

M.I., N.K., R.I., M.T., T.T., M.M., and K.Y. were involved in the data collection. M.I. and K.Y. wrote the manuscript. K.Y. supervised the study. All the authors have read and approved the final manuscript.

## Funding

This work was supported by Kanae Foundation for the Promotion of Medical Science. Pfizer Health Research Foundation. Japan Agency for Medical Research and Development, 21gm6310015h0002, 22gm1510009h0001, 21wm04250XXs0101, 21uk1024002h0002, 23gm1910004s0301, 22wm0525010h0002. Japan Society for the Promotion of Science, 23H04173, 20K15935, 21H05698.

## Ethics Statement

The protocol for this research project has been approved by a suitably constituted Ethics Committee of Nara Medical University (Approval Nos. 1319, 2535) and conforms to the provisions of the Declaration of Helsinki. All informed consent was obtained from the subject(s) and/or guardian(s). This study was conducted in accordance with the Declaration of Helsinki and approved by the Institutional Review Board of Nara Medical University.

## Consent

Written informed consent was obtained from the parents or legal guardians of all participants, and assent was obtained from the children when appropriate.

## Conflicts of Interest

The authors declare no conflicts of interest.

## Supporting information


**Table S1:** Psychiatric comorbidities in the autism spectrum disorder group.
**Table S2:** Psychotropic medication use in the autism spectrum disorder group.

## Data Availability

The data that support the findings of this study are available on request from the corresponding author. The data are not publicly available due to privacy or ethical restrictions.
