# Supplementary material for: Salivary Oxytocin and Socio‐Emotional Functioning in Children and Adolescents With and Without Autism Spectrum Disorder
Source: Neuropsychopharmacol Rep. 2026 Jun 4;46(2):e70138. doi: 10.1002/npr2.70138 (PMC13581109; doi:10.1002/npr2.70138)
Supplement: Supplementary file 1 [file NPR2-46-e70138-s001.docx]

**Supplementary Table S1. Psychiatric comorbidities in the autism spectrum disorder group**

| Comorbid psychiatric condition | n |
| --- | --- |
| Attention-deficit/hyperactivity disorder | 4 |
| Obsessive–compulsive disorder | 1 |
| Tic disorder | 1 |
| Adjustment disorder | 1 |

This table summarizes the psychiatric comorbidities observed in participants with autism spectrum disorder. Values indicate the number of participants diagnosed with each comorbid psychiatric condition.

**Supplementary Table S2. Psychotropic medication use in the autism spectrum disorder group**

| Medication class | n |
| --- | --- |
| Second-generation antipsychotics | 9 |
| Antidepressants | 4 |
| Benzodiazepines or related agents | 4 |
| Attention-deficit/hyperactivity disorder medications | 3 |
| Melatonin receptor agonists | 3 |
| Orexin receptor antagonists | 3 |
| Mood stabilizers / antiepileptic drugs | 2 |
| First-generation antipsychotics | 1 |
| Non-benzodiazepine hypnotics (Z-drugs) | 1 |
